# Theory, Measurement, and Psychometric Properties of Risk and Protective Factors for Drug Misuse Among Adolescents Living on or near the Cherokee Nation Reservation

**DOI:** 10.1007/s42844-023-00112-1

**Published:** 2023-09-25

**Authors:** Melvin D. Livingston, Caroline M. Barry, Ashna Jagtiani, Terrence K. Kominsky, Juli R. Skinner, Bethany J. Livingston, Megan Harmon, Emily A. Ivanich, Hannah L.F. Cooper, Alexander C. Wagenaar, Kelli A. Komro

**Affiliations:** 1 Emory University, Rollins School of Public Health, Department of Behavioral, Social and Health Education Sciences, Atlanta, GA, USA; 2 Cherokee Nation Behavioral Health, Tahlequah, OK, USA; 3 Neighbors Building Neighborhoods Nonprofit Resource Center, Muskogee, OK, USA

**Keywords:** Risk factors, Protective factors, Measures, Psychometrics, Substance misuse, Adolescence, American Indian

## Abstract

A team of tribe-based behavioral health specialists and university-based researchers partnered to implement a cluster randomized trial for the prevention of drug misuse among adolescents attending public high schools on or near the Cherokee Nation Reservation in northeastern Oklahoma. The conceptual framework, which guided intervention and measurement design for the trial, incorporates indigenous knowledge and worldviews with empirically-based frameworks and evidence-based practices. Our goal is to serve multicultural youth, families, and schools and to provide a model of effective strategies for wide dissemination. This paper presents the conceptual model, survey design, and psychometric properties of scales to measure risk and protective factors for substance misuse. The survey includes common measures drawn from the PhenX Toolkit on substance use patterns—adolescent module, measured with standard items from the Monitoring the Future (MTF) study and items harmonized across ten NIH-funded research projects with diverse samples of youth. In our trial, brief (20-minute) self-report questionnaires were administered to 10th grade students in fall 2021 (*n* = 919, 87% response rate) and spring 2022 (*n* = 929, 89% response rate) in 20 participating high schools on or near the Cherokee Nation Reservation. The sample primarily fell into the following three categories of race/ethnicity identification: only American Indian (AI-only, 29%), AI and another race/ethnicity (AI+, 27%), and only White (35%). Results indicate that risk and protective factor scales were reliably and validly measured with 10 scales and 10 subscales. There were minimal differences between youth who identified as AI only, AI+, and White only, especially for the main scales, which provide confidence in the interpretation of trial outcomes across demographic groups. Study results may not be generalizable to AI/AN youth who live and attend school in more homogenous reservation lands, or alternatively, live in large diverse metropolitan areas.

The goal of this paper is to provide evidence of the reliability and validity of standard measures of substance misuse risk and protective factors for use with American Indian youth. The measures were designed to assess important risk and protective factors, as well as substance use and mental health outcomes, among a diverse sample of high school students living on or near the Cherokee Nation Reservation. A team of tribe-based behavioral health specialists and university-based researchers partnered to design and implement a cluster randomized trial for the prevention of substance misuse and promotion of mental health among adolescents. We have been collaborating and building our team’s partnership for over 10 years (Komro et al., 2017; Komro et al., 2015a), with some changes in team members over time (Komro et al., 2022b). Our collaboration has been built on mutual interests in optimal youth development and use of data to test effectiveness of efforts in the real world. Our values were aligned to support underserved youth and families directly with shared expertise and resources, as well as to contribute more broadly through dissemination of scientific findings.

Our trial was part of the Helping to End Addiction Long-Term (HEAL) Prevention Cooperative (HPC), a group of ten research projects and a coordinating center, funded by the National Institutes of Health and administered by the National Institute on Drug Abuse (Komro et al., 2022a; Ridenour et al., 2022). The HPC Coordinating Center at RTI International led efforts for core measure selection and harmonization across sites (Ridenour et al., 2022). Each research project developed a distinct intervention for specific populations and settings to prevent opioid misuse among older adolescents and young adults (Ridenour et al., 2022). In addition to our project in the Cherokee Nation, one additional project focused on American Indian/Alaska Native young adults (Komro et al., 2022b).

The goal of our trial was universal primary prevention of substance misuse and promotion of mental health among a cohort of high school students living on or near the Cherokee Nation Reservation in northeast Oklahoma (Komro et al., 2022a). We recruited 20 rural high schools and randomly assigned them to an intervention or delayed-intervention comparison condition. The Cherokee Nation Institutional Review Board (IRB) served as the IRB of record and approved the trial protocol, as well as each dissemination product. The intervention integrated family, school, and community strategies, based on our previous collaboration (Komro et al., 2017) and included (1) connect—a universal screening and brief motivational interviewing intervention within schools and (2) family action kits mailed directly to homes, with corresponding school and community dissemination.

In order to optimize cultural acceptability and intervention effectiveness, we relied on a collaborative approach between Cherokee Nation Behavioral Health specialists and prevention science expertise of university-based team members (Komro et al., 2022b). We designed a working conceptual model incorporating indigenous knowledge and worldviews with western frameworks and evidence-based practices. Our goal was to serve multicultural youth, families, and schools within and near the Cherokee Nation Reservation and to provide a model for community-based primary prevention for wide dissemination.

We first present our integrated conceptual framework, which guided intervention and measurement design. We then describe measures used to test the effectiveness of the intervention and an examination of how well the measures perform among a large sample of rural American Indian youth.

## Conceptual Framework

The conceptual framework guided selection of intervention objectives and measures and was based on merging socio-ecological and risk and protective frameworks (Hawkins et al., 1992; Keyes et al., 2014; Komro et al., 2016; Wagenaar & Perry, 1994) with an indigenous relational worldview perspective (Blackstock, 2019; Cross, 2007). The integrated conceptual framework is meant to highlight dynamic, multilevel, inter-relationships between contextual (i.e., societal, community, social) and intra- and inter-level (i.e., mind, body, spirit) factors that influence health and well-being as it relates to abstinence or initiation and escalation of substance misuse during adolescence into young adulthood.

## Method

### Participants

We recruited 20 rural high schools on or near the Cherokee Nation Reservation and randomly assigned them to an intervention or delayed-intervention comparison condition. In fall 2021, following Cherokee Nation IRB approved parent consent and student assent procedures, we enrolled and surveyed a cohort of 10th-grade students with the first follow-up survey conducted in spring 2022.

### Measures

The survey was designed to measure risk and protective factors at the individual to community levels as depicted in the conceptual model. The survey included common measures that we have used in previous studies (Komro et al., 2015b) and drawn from the PhenX Toolkit on substance use patterns—adolescent module (module #510301), measured with standard items from the Monitoring the Future (MTF) study (https://monitoringthefuture.org). PhenX uses a consensus process and inputs from the scientific community to provide well-established, high-quality, low-burden measurement protocols (https://www.phenx.org/).

Primary outcome measures included frequency (number of days) during the past 30 days of any: (1) alcohol use, (2) heavy alcohol use (defined as having at least four, among young women and those not disclosing gender, or five, among young men, standard alcoholic drinks within a couple of hours), (3) marijuana use, and (4) prescription opioid misuse (defined as “without a doctor’s prescription or differently than how a doctor or medical provider told you to use it”).

Measures of substance misuse-related problems included pain, depression, and anxiety. Pain was measured with the 4-item PROMIS Pediatric Pain Interference Scale (Cunningham et al., 2017; Varni et al., 2010). Depression was measured with the 8-item Patient Health Questionnaire depression scale (PHQ-8), established as a valid measure of current depression in the general population (Kroenke et al., 2009). Anxiety was measured with the Generalized Anxiety Disorder 7-item scale (GAD-7), found to have acceptable specificity and sensitivity and to differentiate between mild and moderate GAD among adolescents (Mossman et al., 2017).

Key risk and protective factors (and hypothesized mediators for intervention effects) included social support, perceived availability of drugs, social normative disapproval beliefs, self-efficacy, perceptions of getting in trouble for use, and normative estimates. Social support from community members, parents/caregivers, teachers, and friends were adapted from the School Support Scale (Hanson & Kim, 2007) and assessed with 24 items which are responded to on a 4-point scale where 0 = never and 3 = often. Adapted from the PhenX MTF items, ease or difficulty in accessing alcohol, marijuana, and prescription opioids was assessed with 12 items using a 4-point scale where 0 = very difficult to get and 3 = very easy to get. Adapted from MTF, participants were asked 12 items to assess if they think various social groups disapprove of young people drinking alcohol, using marijuana, and prescription opioid misuse (parents, community adults, peers, self). Responses were 0 = do not disapprove, 1 = disapprove, and 2 = strongly disapprove. Self-efficacy was assessed with 4 items asking how easy or hard it would be for participants to ask for help or refuse alcohol or drugs (Choi et al., 2013; Komro et al., 2015b). Responses were a 4-point scale, where 0 = very easy and 3 = very hard. Adapted from MTF, perceptions of getting into trouble with caregivers, teachers, or police for substance use (alcohol, marijuana, prescription opioid misuse) was measured with three items with response options of 0 = very little chance, 1 = little chance, 2 = some chance, and 3 = very good chance. Adapted from the PhenX Communities That Care items (Arthur et al., 2007), normative estimates of peer drug use (alcohol, marijuana, prescription opioid misuse) were assessed with 3 items asking how many of their peers in school used drugs in the past year. Possible responses were 0 = none or almost none, 1 = less than half, 2 = about half, 3 = more than half, and 4 = almost all or all. Tribal identity was measured with adaptations of three items from the 6-item Multigroup Ethnic Identity Measure–Revised (MEIM-R) (Phinney, 1992). Students who self-identified as American Indian/Alaska Native were asked three questions on a 5-point scale from strongly disagree to strongly agree: (1) I have a strong sense of belonging to my tribe, (2) I understand pretty well what my tribal identity means to me, and (3) I feel a strong attachment towards my tribe.

### Survey Procedure

Brief (20 minute) self-report questionnaires were administered to 10th-grade students in fall 2021 (wave 1) and spring 2022 (wave 2) in 20 participating high schools. The response rate was 87% with 919 completed surveys in the fall 2021 survey, and 89% with 929 completed surveys in spring 2022. Reasons for nonresponse in order of frequency included (1) nonresponse from remote (i.e., off-site) students, (2) parent refusals, (3) parent consent undeliverable, (4) student absences, (5) student refusals, and (6) alternative education or vocational/technology students who were unable to be surveyed in school.

Nearly half of the sample identified as female (48%), nearly half as male (48%), and 4% selected “decline to answer.” The mean age was 15.5 years in fall 2021. The study sample is primarily American Indian (AI) and White. For the race/ethnicity item, which instructs participants to select all that apply, 28.9% reported being only AI, 26.7% reported being AI and another race/ethnicity (AI+), 35.1% reported being only White, and 9.2% reported being another racial/ethnic category.

### Psychometric Analysis

We assessed reliability and validity of 10 scales, and a further 10 associated subscales, measuring risk and protective factors associated with substance use among those students identifying as AI only, AI+, or White only (*n* = 834). Specifically, we evaluated factor structure, concurrent validity, and predictive validity of each scale. We further evaluated measurement invariance across three AI identity categories. Finally, we tested differences in validity estimates by each AI identity group. The tribal identity scale was not used in validity analyses due to a lack of available appropriate criterion measures but was retained for all analyses of factor structure and invariance.

#### Factor Structure

We assessed the fit of the hypothesized factor structure for each scale using confirmatory factor analyses (CFA) using wave 1 survey data. Each scale was assessed in separate CFA measurement models. For models containing multiple subscales (social support, perceived substance use norms, and perceived substance use access), both first and second order CFA models were evaluated. Models were identified by standardizing the latent factor. To account for the ordinal nature of our Likert indicators, all CFA models were estimated using diagonally weighted least squares with mean and variance corrections (WLSMV). Model fit was assessed by both an inspection of factor loadings and the use of fit statistics. Fit statistics used included the comparative fit index (CFI), the root mean square error of approximation (RMSEA), and the standardized root mean square residual (SRMR). To account for the mean and variance corrections used in our WLSMV estimation, robust CFI and RMSEA statistics were used. CFI values greater than 0.90 indicate reasonably good model fit. RMSEA values less than or equal to 0.05 indicate close approximate fit; values between 0.05 and 0.08 suggest reasonable fit, and values greater than or equal to 0.10 suggest poor model fit. SRMR values of less than 0.08 indicate reasonable model fit. Notably, some of our single factor CFA models contain only three indicators which will lead our fit statistics to indicate perfect fit regardless of the underlying structure. For these models, factor loadings are still informative, and they are retained in all reported analyses for consistency with subsequent measurement invariance testing.

Measurement invariance testing was carried out using multi-group CFA models. We began by evaluating a simple model assuming configural invariance. Specifically, we estimated a model with the same underlying factor structure for all three identity groups, while allowing the factor loadings and indicator intercepts to freely vary across groups. When configural invariance was indicated, we then proceeded to test for weak invariance by holding the factor loadings equivalent across each of the three identity groups. When weak invariance was indicated, we then proceeded to test for strong invariance by holding both the factor loadings and intercepts equivalent across the identity groups. Strict invariance was not assessed due to guidance provided in both Little (2013) and Kline (2015) regarding the impact of random measurement error on residual error variance. To establish configural invariance, overall fit of the unrestricted multi-group CFA models were assessed similarly to the previously described single-group CFA models. For three item CFA models, configural variance is assumed given the lack of available degrees of freedom. To assess whether subsequent levels of invariance were met, we assessed changes in model fit with the additional group restrictions based on a change in the robust CFI statistic. A difference in the robust CFI statistic between the more restrictive and free models of less than 0.01 was considered adequate to establish invariance. All measurement invariance analyses were carried out using the “lavaan” package in R version 4.1.0.

#### Validity

**Fig. 1:**
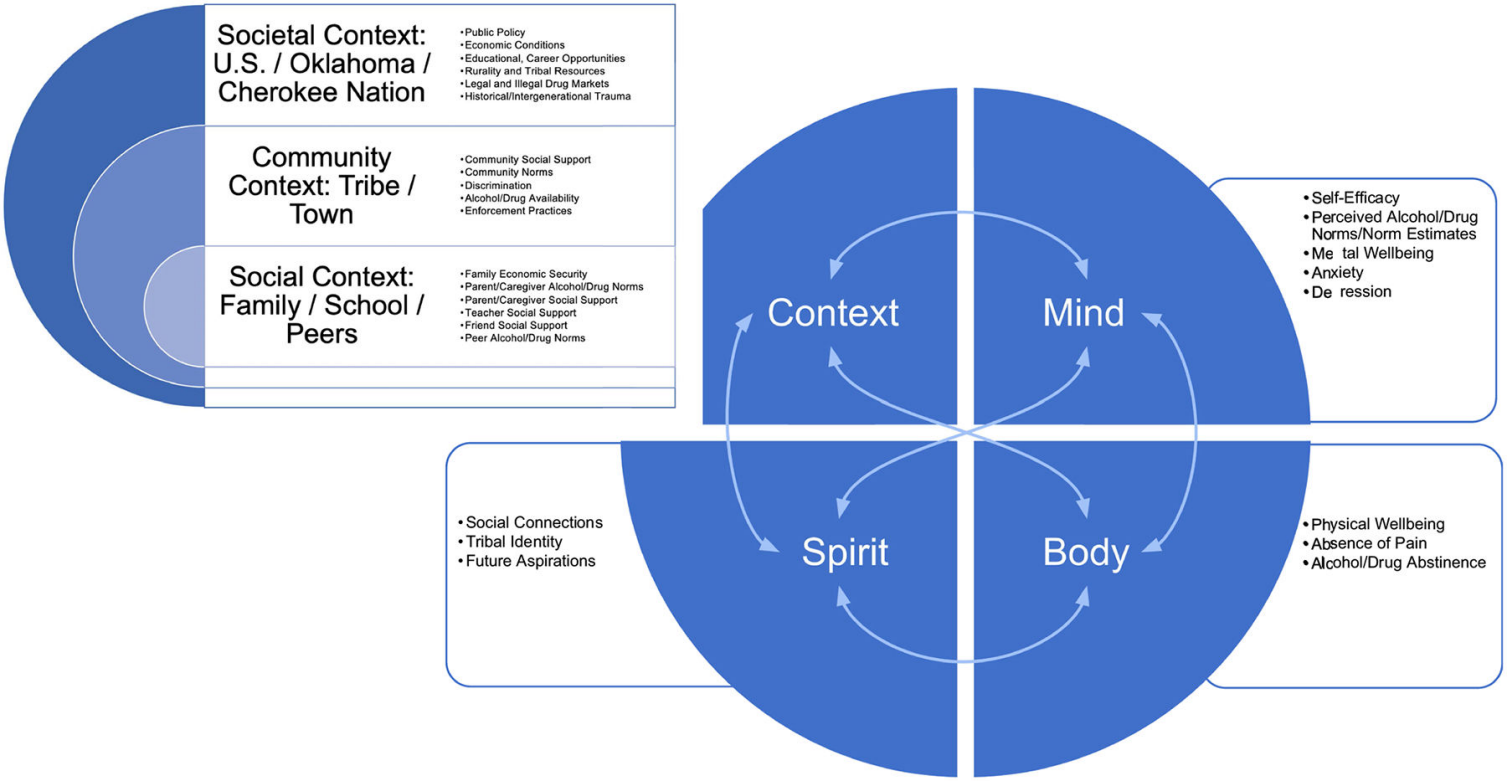
Conceptual framework merging indigenous relational wordview and socio-ecological risk and protective factors for youth substance misuse

Concurrent validity was estimated using the cross-sectional models at wave 1 of each scale with an applicable substance use outcome; predictive validity was estimated using each scale at wave 1 and the substance use outcome at wave 2. While no gold-standard is available in our data for the scales, we establish validity by estimating the relationship between the scales and a substance use criterion based on well-established behavioral theory, as presented in Fig. 1. To estimate validity, the majority of scales used an overall substance use criterion calculated by a participant indicating either past 30-day use of alcohol, marijuana, or opioids. For scales that were specific to a given substance, reports of past 30-day use of that specific substance were used as the criterion (e.g., marijuana use norms used past 30-day marijuana use for validity estimates). Odds ratios for the association between each scale and criterion were then estimated using mixed effects logistic regression models with a random intercept for study school. Wave 1 scales were standardized in each model to allow for easy comparisons for validity estimates across each scale.

Differences in concurrent and predictive validity were estimated using mixed effects logistic regressions similar to validity estimates from the full sample. These models contained an interaction between the scale and AI identity group indicators. Odds ratios were then estimated for each identity group, and an *F*-test was used to test the statistical significance of the scale by AI identity group interaction. All models were estimated using “glmer” in R version 4.1.0.

## Results

### Substance Use Indices

Table 1 presents descriptive statistics for planned primary outcomes, scales for secondary outcomes, and scales for other substance use related problems. All descriptive results are presented at wave 1 for the full sample and by AI identity.

### Factor Structure

Confirmatory factor analysis was conducted to assess factor loadings, model fit indices, and overall reliability of each scale and subscale. Factor loadings and fit statistics for first order models can be found in Table 2. For scales measuring overall social support, perceived substance use access, and normative disapproval beliefs, no first order model was found to fit well. A second order CFA model was found to fit overall social support well (robust CFI = 0.996, robust RMSEA = 0.021, SRMR = 0.037), but not for perceived substance use access or normative disapproval beliefs. As a result, scales for overall perceived substance use access and normative disapproval beliefs were abandoned in favor of their substance-specific subscales in all remaining analyses.

Across the remaining first order CFA models for scales and subscales, there was a consistent pattern of well-fitting models (Table 2). With factor loadings ranging from 0.694 to 0.859, the pain scale items demonstrated strong associations with the latent construct. This suggests that each item effectively measures the concept of pain, contributing to the overall reliability of the scale. The model demonstrated an excellent fit to the data, as evidenced by a robust CFI of 0.99, a robust RMSEA of 0.046, and a robust SRMR of 0.027. The PHQ-8 and GAD-7 both had strong model fit statistics lending support for their use as measures of depressive symptoms and anxiety symptoms among this population. Factor loadings for the PHQ-8 and GAD-7 ranged from 0.645 to 0.786 and 0.624 to 0.864, respectively, with robust CFIs greater than 0.99 and robust RMSEA and robust SRMR values indicating close approximate fit on both scales. Scales measuring source specific social support from parents/caregivers, teachers, friends, and other adults in the community were tested using CFA on the subscale for each source. Results showed strong factor loadings ranging from 0.648 to 0.932 across subscales, with robust CFIs all greater than 0.99. All robust RMSEA values were below the cutoff for close approximate fit, and robust SRMRs reflect reasonable model fit across social support subscales. Similarly, scales measuring perceived access to alcohol, marijuana, and prescription opioids also had factor loadings and model fit statistics that lend support for their use as measures of the desired latent constructs among these youth. Factor loadings for alcohol access, marijuana access, and prescription opioid access were all high, ranging from 0.547 to 0.794, 0.515 to 0.827, and 0.545 to 0.862, respectively. Robust CFIs were greater than 0.99 for all three perceived access scales, reflecting excellent fit. Robust RMSEAs indicate close approximate fit with values less than 0.05 for alcohol and prescription opioid access and reasonable fit for marijuana access at 0.065. Robust SRMRs were similarly low (0.027–0.05). Items on the normative disapproval beliefs scales for alcohol, marijuana, and prescription opioids had generally strong factor loadings (alcohol: 0.489–0.671; marijuana: 0.497–0.770; prescription opioids: 0.463–0.747) and CFA model fit statistics. Robust CFIs across substance-specific scales were all greater than 0.98, robust SRMRs less than 0.05, and robust RMSEAs within (or close to, see marijuana robust RMSEA of 0.091) range for reasonable fit. The remaining scales (self-efficacy to refuse, perception of getting in trouble, normative estimates of peer use, and tribal identity) had strong factor loadings, but model fit could not be assessed given that these models were just identified.

Formal invariance testing was carried out for all scales and subscales with the exception of overall social support, overall perceived substance use access, and normative disapproval beliefs. Measurement invariance for overall social support was not assessed due to lack of convergence in the 2nd order configural model. Measurement invariance was not assessed for overall perceived substance use access and normative disapproval beliefs due to lack of a well-fitting model in the full sample. All remaining scales were found to be strongly invariant based on the criterion that the change in robust CFI across more restrictive models did not exceed 0.01 (Table 3). Additionally, all fit statistics for weak and strong invariance models indicated good fit with all robust CFIs in excess of 0.95, robust RMSEAs below 0.08, and all SRMRs below 0.08 (Appendix 1).

### Validity

#### Concurrent Validity

Table 4 presents overall and by group concurrent validity estimates for each scale using cross-sectional models at wave 1 Overall, every standard deviation increase in pain, depression, and anxiety scales was associated with 1.35, 1.55, and 1.38 times the odds of overall substance use. Additionally, every standard deviation increase in overall perceived substance use access was associated with 2.3 times the odds of overall substance use. Similar results were found for all three substance-specific subscales with the highest odds with marijuana use (3.41). Also, every standard deviation increase in normative estimates of peer alcohol and drug use was associated with 1.92 times the odds of overall substance use. Conversely, every standard deviation increase in overall social support was associated with 0.68 times the odds of overall substance use. Similar results were found in all three subscales of social support with the lowest odds of overall substance use with parent/caregiver support (0.63). Similarly, every standard deviation increase in overall normative disapproval beliefs was associated with 0.44 times the odds of overall substance use. Similar results were found for all three substance-specific subscales with the lowest odds with marijuana use (0.28). Finally, every standard deviation increase in self-efficacy to refuse alcohol and drugs and the perception of getting in trouble for substance use was associated with 0.43 and 0.52 times the odds of overall substance use.

Concurrent validity differed significantly based on the *F*-test by identity groups in one scale and one subscale. The association between self-efficacy to refuse alcohol and drugs and overall substance use differed significantly between identity groups (*p* < 0.05) where AI+ youth had the lowest odds (0.23) of overall substance use with every standard deviation increase in the self-efficacy to refuse alcohol and drugs scale. The association between adult support and overall substance use also differed significantly between identity groups (*p* < 0.05) where White youth had the lowest odds (0.60) of overall substance use with every standard deviation increase in adult support.

#### Predictive Validity

Table 5 presents overall and by group predictive validity estimates using each scale at wave 1 and the substance use outcome at wave 2. Predictive validity estimates reflect similar associations in the same direction as observed for concurrent validity. Overall, every standard deviation increase in pain, depression, and anxiety scales at wave 1 was associated with 1.41, 1.74, and 1.61 times the odds of overall substance use, i.e., either past 30-day use of alcohol, marijuana, or opioids at wave 2. Additionally, every standard deviation increase in overall perceived substance use access at wave 1 was associated with 1.97 times the odds of overall substance use at wave 2. Similar results were found for all 3 substance-specific subscales with the highest odds with marijuana use (2.52). Also, every standard deviation increase in normative estimates of peer alcohol and drug use at wave 1 was associated with 1.50 times the odds of overall substance use at wave 2. Conversely, every standard deviation increase in overall social support at wave 1 was associated with 0.61 times the odds of overall substance use at wave 2 Similar results were found in all 3 subscales of social support with the lowest odds of overall substance use with parent/caregiver support (0.64). Similarly, every standard deviation increase in overall normative disapproval beliefs at wave 1 was associated with 0.54 times the odds of overall substance use at wave 2. Similar results were found for all three substance-specific subscales with the lowest odds with marijuana use (0.29). Finally, every standard deviation increase in self-efficacy to refuse alcohol and drugs and the perception of getting in trouble for substance use at wave 1 was associated with 0.54 and 0.59 times the odds of overall substance use at wave 2.

Predictive validity differed significantly based on the *F*-test by identity groups in only one scale. The association between self-efficacy to refuse alcohol and drugs at wave 1 and overall substance use at wave 2 differed significantly between identity groups (*p* < 0.05) where AI+ youth had the lowest odds (0.34) of overall substance use at wave 2 with every standard deviation increase in self-efficacy to refuse alcohol and drugs at wave 1.

## Discussion

The multi-item scales to measure risk and protective factors targeted by our multilevel preventive intervention performed well among AI and White youth attending high schools on or near the Cherokee Nation Reservation. Factor analyses in the overall sample demonstrated adequate model fit across almost all scales, and item loadings indicated that our measured items reasonably measure the proposed constructs. Results from the overall sample support the use of these scales in planned intervention evaluation analyses for the parent trial. Notably, these scales exhibited remarkable measurement invariance across AI identity groups in our sample, supporting their use in comparing potential intervention effects by AI status.

Concurrent and predictive validity was also evident for each scale based on correspondence between our criterion results and those predicted by our theoretical framework. For criterion validity, all but one of our scales and subscales were significantly associated with substance use in the theoretically aligned direction as risk or protective factors. Social support from friends was not associated with reductions in substance use and may indicate heterogeneous effects based on peer substance use norms. Two of our scales exhibited differential concurrent validity across AI identity groups. Adult social support was protective for White youth, but not AI only and AI+ groups. While self-efficacy to refuse alcohol and drugs was strongly associated with reductions in substance use for all groups, this association was stronger among AI+ youth. Similarly, the majority of predictive validity estimates were significant and in the theorized direction. Notably, the associations between both perceived access to prescription opioids and normative disapproval beliefs for prescription opioids with our opioid use criterion were attenuated in predictive validity models and no longer statistically significant. Only self-efficacy to refuse drugs and alcohol exhibited differential predictive validity across AI identity groups, with patterns similar to those observed in concurrent validity models.

Overall, all our scales performed well for the full sample and for the three subgroups defined by identity, providing support for their use in measuring changes in risk and protective factors over time and for measurement of effectiveness of the multilevel preventive intervention.

### Limitations and Future Directions

The results of this study may not generalize outside the context of the ongoing trial due to the uniqueness of this study’s sample of AI youth. With forced removal of Cherokee people from their once vast ancestral lands in what is now the southeastern US, to Indian country, in what is now the State of Oklahoma, jurisdictional boundaries of the Cherokee Nation were set. However, when Oklahoma became a state in 1907, the tribe’s land was allotted to individual land owners, with much of the land quickly acquired by non-Indians (for additional historical details, see https://www.cherokee.org/about-the-nation/history/). Consequently, the land within the 14-county jurisdictional boundaries of the Cherokee Nation Reservation is primarily owned by non-Indians with the geographic area being racially mixed but majority White, as is evident by the demographic characteristics of the study sample. Therefore, our study results may not be generalizable to AI/AN youth who live and attend school in more homogenous reservation lands, or alternatively, live in large diverse metropolitan areas. In future research with either more homogenous or diverse samples, there may be a need to adapt measures.

Despite limitations, results provide confidence in the use of this brief survey instrument to reliably and validly measure targeted risk and protective factors and for outcome assessments of preventive interventions. The comprehensive survey was completed within 20 minutes, with approximately 30 minutes of class time used for complete survey administration. The survey included core measures of substance use, pain, depression, and anxiety that were harmonized across ten research projects as part of the HEAL prevention initiative, which will facilitate even greater understanding of developmental trajectories and intervention effectiveness across various distinct populations of adolescents and young adults.

## Conclusion

Our conceptual framework, which guided selection of intervention objectives and measures, merged socio-ecological and risk and protective frameworks (Hawkins et al., 1992; Keyes et al., 2014; Komro et al., 2016; Wagenaar & Perry, 1994) with an indigenous relational worldview perspective (Blackstock, 2019; Cross, 2007). The majority of targeted risk and protective factors were deemed reliably and validly measured across our 10 scales and 10 subscales included in a brief 20-minute survey. While measures of perceived substance use access and normative disapproval beliefs aggregated across alcohol, marijuana, and prescription opioid misuse were not able to be validated, substance-specific subscale performed well. Factor analysis demonstrated strong invariance of validated scale across youth who identified as AI-only, AI and another race/ethnicity, and White-only providing confidence in the use of these scales across demographic groups. Observed differences in criterion and predictive validity of each scale were also minimal across race/ethnicity further reinforcing the use of these scales in our heterogeneous study population.

## Supplementary Material

ESM1

## Figures and Tables

**Table 1:** Baseline descriptive statistics for scales used for the overall sample and by AI identity

Scale	Range	Overall mean (SD) (*N* = 834)	White mean (SD) (*N* = 323)	AI+ other mean (SD) (*N* = 245)	AI only mean (SD) (*N* = 266)
Pain^a^	1–5	1.96 (0.99)	1.95 (0.98)	1.95 (0.99)	1.97 (1.02)
Depression (PHQ-8)	0–24	7.42 (6.02)	7.14 (5.83)	7.45 (5.91)	7.73 (6.34)
Anxiety (GAD-7)	0–21	6.68 (5.81)	6.49 (5.68)	6.75 (5.67)	6.86 (6.10)
Social support—overall^a^	1–4	3.36 (0.55)	3.40 (0.53)	3.35 (0.53)	3.34 (0.58)
Parent/caregiver^a^	1–4	3.55 (0.57)	3.59 (0.50)	3.54 (0.54)	3.52 (0.66)
Friend^a^	1–4	3.40 (0.70)	3.42 (0.70)	3.40 (0.65)	3.38 (0.74)
Teacher^a^	1–4	3.33 (0.76)	3.34 (0.74)	3.36 (0.73)	3.30 (0.81)
Adult (community)^a^	1–4	3.17 (0.94)	3.24 (0.90)	3.12 (0.94)	3.14 (0.98)
Perceived substance use access—overall^a^	1–4	1.76 (0.64)	1.77 (0.64)	1.82 (0.62)	1.71 (0.65)
Alcohol^a^	1–4	2.01 (0.78)	2.02 (0.79)	2.06 (0.73)	1.96 (0.81)
Marijuana^a^	1–4	1.87 (0.81)	1.85 (0.81)	1.92 (0.80)	1.85 (0.82)
RX opioids^a^	1–4	1.41 (0.61)	1.42 (0.60)	1.47 (0.64)	1.33 (0.61)
Normative disapproval beliefs—overall^a^	1–3	2.28 (0.43)	2.31 (0.40)	2.27 (0.43)	2.26 (0.46)
Alcohol^a^	1–3	2.14 (0.51)	2.16 (0.48)	2.11 (0.51)	2.13 (0.54)
Marijuana^a^	1–3	2.19 (0.55)	2.23 (0.52)	2.17 (0.56)	2.15 (0.56)
RX opioids^a^	1–3	2.53 (0.44)	2.55 (0.40)	2.54 (0.45)	2.50 (0.49)
Self-efficacy to refuse alcohol and drugs^a^	1–4	3.45 (0.74)	3.49 (0.74)	3.48 (0.66)	3.37 (0.82)
Perception of getting in trouble for alcohol and drug use^a^	1–4	3.50 (0.76)	3.53 (0.72)	3.53 (0.70)	3.44 (0.85)
Normative estimates of peer alcohol and drug use^a^	1–5	2.42 (0.85)	2.44 (0.85)	2.47 (0.85)	2.34 (0.84)
Tribal identity^ab^	1–5			3.04 (0.99)	3.33 (1.14)

*PHQ-8* Patient Health Questionnaire depression scale-8, *GAD-7* Generalized Anxiety Disorder Scale-7, *RX* prescription

a Scale coded as the mean of constituent Likert items

b Only asked to those reporting as AI (American Indian) or AI+ other

**Table 2:** Confirmatory Factor Analysis (CFA)

Scale	Factor loadings	Robust CFI	Robust RMSEA	Robust SRMR
Pain		0.999	0.046	0.027
Trouble sleeping	0.806			
Felt sad	0.767			
Hard to pay attention	0.859			
Hard to stay standing	0.694			
Depression (PHQ-8)		0.994	0.041	0.039
Little interest or pleasure in doing things	0.684			
Feeling down, depressed, or hopeless	0.786			
Trouble falling or staying asleep, or sleeping too much	0.703			
Feeling tired or having little energy	0.755			
Poor appetite or overeating	0.677			
Feeling bad about yourself	0.717			
Trouble concentrating on things	0.648			
Moving or speaking so slowly/being so fidgety or restless	0.645			
Anxiety (GAD-7)		0.997	0.034	0.030
Feeling nervous, anxious or on edge	0.816			
Not being able to stop or control worrying	0.864			
Worrying too much about different things	0.857			
Trouble relaxing	0.821			
Being so restless that it is hard to sit still	0.624			
Becoming easily annoyed or irritable	0.655			
Feeling afraid as if something awful might happen	0.751			
Parent/caregiver support		0.991	0.039	0.054
Really cares about me	0.680			
Tells me when I do a good job	0.801			
Notices when I am not there	0.648			
Always wants me to do my best	0.658			
Listens to me when I have something to say	0.792			
Believes that I will be a success	0.783			
Teacher support		1.000	0.012	0.017
Really cares about me	0.864			
Tells me when I do a good job	0.830			
Notices when I am not there	0.766			
Always wants me to do my best	0.830			
Listens to me when I have something to say	0.808			
Believes that I will be a success	0.869			
Friend support		1.000	0.004	0.012
Really cares about me	0.786			
Tells me when I do a good job	0.779			
Notices when I am not there	0.772			
Always wants me to do my best	0.846			
Listens to me when I have something to say	0.772			
Believes that I will be a success	0.844			
Adult (community) support		1.000	0.013	0.013
Really cares about me	0.902			
Tells me when I do a good job	0.901			
Notices when I am not there	0.876			
Always wants me to do my best	0.932			
Listens to me when I have something to say	0.901			
Believes that I will be a success	0.924			
Perceived alcohol access		0.996	0.047	0.027
From the place where you live	0.547			
From a store	0.569			
From someone your age	0.724			
From an older friend or another adult	0.794			
Perceived marijuana access		0.993	0.065	0.050
From the place where you live	0.515			
From a dispensary without a doctor’s RX	0.523			
From a dealer	0.797			
From someone your age	0.827			
From an older friend or another adult	0.807			
Perceived RX opioid access		0.996	0.041	0.049
From the place where you live	0.545			
From a dispensary without a doctor’s RX	0.595			
From a dealer	0.791			
From someone your age	0.862			
From an older friend or another adult	0.837			
Alcohol normative disapproval beliefs		0.989	0.068	0.031
Self	0.671			
People your age	0.489			
Adults	0.630			
Parent(s)/caregiver(s)	0.666			
Marijuana normative disapproval beliefs		0.984	0.091	0.041
Self	0.770			
People your age	0.497			
Adults	0.620			
Parent(s)/caregiver(s)	0.679			
RX opioid normative disapproval beliefs		0.988	0.065	0.041
Self	0.652			
People your age	0.463			
Adults	0.747			
Parent(s)/caregiver(s)	0.663			
Self-efficacy to refuse alcohol and drugs		1.000	0.000	0.000
When you are feeling down	0.759			
When you are celebrating or at a party	0.960			
When offered by a friend	0.705			
Perception of getting in trouble for alcohol and drug use		1.000	0.000	0.000
Alcohol	0.768			
Marijuana	0.843			
RX opioid	0.733			
Normative estimates of peer alcohol and drug use		1.000	0.000	0.000
Alcohol	0.771			
Marijuana	0.810			
RX opioid	0.539			
Tribal identity		1.000	0.000	0.000
Strong sense of belonging to my tribe	0.909			
Understand pretty well what my tribal identity means to me	0.897			
Feel a strong attachment towards my tribe	0.930			

*CFI* comparative fit index, *RMSEA* root mean square error of approximation, *SRMR* standardized root mean square residual, *GAD-7* Generalized Anxiety Disorder Scale-7, *PHQ-8* Patient Health Questionnaire depression scale-8, *RX* prescription

**Table 3:** Measurement invariance

Scale		Configural invariance CFI	Weak invariance CFI	Strong invariance CFI	Delta CFI weak invariance	Delta CFI strong invariance
Pain		1.000	1.000	1.000	0.000	0.000
Depression (PHQ-8)		1.000	1.000	1.000	0.000	0.000
Anxiety (GAD-7)		0.997	0.997	0.997	0.000	0.000
Social support—overall		a	a	a	a	a
	Parent/caregiver support	0.990	0.977	0.971	0.013	0.006
	Friend support	1.000	1.000	1.000	0.000	0.000
	Teacher support	1.000	1.000	1.000	0.000	0.000
	Adult (community) support	0.999	0.999	0.999	0.000	0.000
Perceived substance use access—overall		b	b	b	b	b
	Perceived alcohol access	1.000	1.000	1.000	0.000	0.000
	Perceived marijuana access	0.995	0.997	0.999	−0.002	−0.002
	Perceived RX opioid access	1.000	1.000	1.000	0.000	0.000
Normative disapproval beliefs—overall		b	b	b	b	b
	Alcohol normative disapproval beliefs	0.994	0.997	0.992	−0.003	0.005
	Marijuana normative disapproval beliefs	1.000	1.000	1.000	0.000	0.000
	RX opioid normative disapproval beliefs	0.994	0.999	1.000	−0.005	−0.001
Self-efficacy to refuse alcohol and drugs		1.000	1.000	1.000	0.000	0.000
Perception of getting in trouble for alcohol and drug use		1.000	1.000	1.000	0.000	0.000
Normative estimates of peer alcohol and drug use		1.000	0.999	0.999	0.001	0.000
Tribal identity		1.000	0.999	0.999	0.001	0.000

a: Invariance not assessed due to lack of convergence in configural invariance model

b: invariance not assessed due to poor model fit in the full sample

*CFI* comparative fit index, *PHQ-8* Patient Health Questionnaire depression scale-8, *GAD-7* Generalized Anxiety Disorder Scale-7, *RX* prescription

**Table 4:** Overall and by AI identity group concurrent validity estimates for each scale at wave 1

Scale	Overall criterion OR (95%)	Criterion OR (95%)— White	Criterion OR (95%)— AI+O	Criterion OR (95%)—AI only	*p*-values
Pain	1.35 (1.16, 1.57)	1.29 (1.00, 1.67)	1.23 (0.94, 1.62)	1.55 (1.19, 2.02)	0.456
Depression (PHQ-8)	1.55 (1.32, 1.81)	1.47 (1.13, 1.92)	1.48 (1.11, 1.97)	1.67 (1.28, 2.18)	0.768
Anxiety (GAD-7)	1.38 (1.19, 1.61)	1.26 (0.97, 1.63)	1.39 (1.05, 1.85)	1.51 (1.16, 1.96)	0.635
Social support—overall	0.68 (0.58, 0.79)	a	a	a	
Parent/caregiver	0.63 (0.54, 0.73)	0.49 (0.37, 0.66)	0.67 (0.50, 0.88)	0.71 (0.57, 0.89)	0.146
Friend	0.94 (0.81, 1.09)	0.90 (0.70, 1.15)	0.93 (0.69, 1.25)	1.00 (0.77, 1.29)	0.846
Teacher	0.73 (0.63, 0.84)	0.57 (0.44, 0.74)	0.88 (0.66, 1.17)	0.78 (0.61, 1.00)	0.068
Adult (community)	0.77 (0.66, 0.89)	0.60 (0.46, 0.78)	0.98 (0.74, 1.30)	0.80 (0.62, 1.03)	**0.037**
Perceived substance use access—overall	b	b	b	b	
Alcohol	2.30 (1.89, 2.80)	2.19 (1.58, 3.02)	2.66 (1.82, 3.89)	2.18 (1.58, 3.02)	0.678
Marijuana	3.41 (2.68, 4.34)	3.52 (2.32, 5.34)	4.46 (2.75, 7.24)	2.75 (1.90, 3.98)	0.284
RX opioids	2.34 (1.78, 3.09)	1.96 (1.17, 3.29)	2.58 (1.50, 4.42)	2.48 (1.62, 3.78)	0.728
Normative disapproval beliefs—overall	b	b	b	b	
Alcohol	0.40 (0.32, 0.49)	0.49 (0.34, 0.70)	0.28 (0.18, 0.42)	0.45 (0.32, 0.63)	0.095
Marijuana	0.28 (0.22, 0.36)	0.24 (0.15, 0.38)	0.23 (0.14, 0.37)	0.37 (0.25, 0.55)	0.200
RX opioids	0.54 (0.40, 0.73)	0.47 (0.26, 0.86)	0.66 (0.38, 1.16)	0.52 (0.33, 0.81)	0.699
Self-efficacy to refuse alcohol and drugs	0.43 (0.36, 0.50)	0.45 (0.34, 0.58)	0.23 (0.15, 0.35)	0.54 (0.42, 0.69)	**0.003**
Perception of getting in trouble for alcohol and drug use	0.52 (0.45, 0.61)	0.52 (0.40, 0.67)	0.46 (0.33, 0.64)	0.56 (0.44, 0.71)	0.655
Normative estimates of peer alcohol and drug use	1.92 (1.61, 2.28)	1.77 (1.33, 2.36)	2.20 (1.59, 3.04)	1.86 (1.37, 2.53)	0.604

a: Group specific ORs were not estimated due to lack of confirmation of scale invariance

b: overall and group specific ORs not estimated due to poor model fit in the overall sample

*p*-values in bold: < 0.05

*PHQ-8* Patient Health Questionnaire depression scale-8, *GAD-7* Generalized Anxiety Disorder Scale-7, *RX* prescription

**Table 5:** Overall and by AI identity group predictive validity estimates using each scale at wave 1 and substance use outcomes at wave 2

Scale	Overall criterion estimate	Criterion estimate— White	Criterion estimate— AI+O	Criterion estimate—AI only	*p*-values
Pain	1.41 (1.19, 1.67)	1.47 (1.10, 1.95)	1.36 (1.02, 1.83)	1.40 (1.04, 1.88)	0.937
Depression (PHQ-8)	1.74 (1.46, 2.07)	1.87 (1.38, 2.52)	1.74 (1.25, 2.42)	1.62 (1.22, 2.16)	0.805
Anxiety (GAD-7)	1.61 (1.36, 1.90)	1.56 (1.18, 2.07)	1.60 (1.17, 2.19)	1.66 (1.25, 2.21)	0.955
Social support—overall	0.61 (0.51, 0.72)	a	a	a	
Parent/caregiver	0.64 (0.54, 0.75)	0.62 (0.45, 0.84)	0.60 (0.43, 0.82)	0.69 (0.54, 0.88)	0.738
Friend	0.81 (0.69, 0.96)	0.92 (0.70, 1.22)	0.77 (0.56, 1.06)	0.74 (0.56, 0.97)	0.501
Teacher	0.66 (0.56, 0.78)	0.57 (0.43, 0.76)	0.68 (0.50, 0.94)	0.74 (0.56, 0.97)	0.445
Adult (community)	0.75 (0.63, 0.88)	0.81 (0.61, 1.07)	0.74 (0.55, 0.99)	0.71 (0.54, 0.93)	0.795
Perceived substance use access—overall	b	b	b	b	
Alcohol	2.02 (1.63, 2.51)	2.24 (1.55, 3.23)	2.20 (1.46, 3.31)	1.75 (1.24, 2.46)	0.566
Marijuana	2.52 (2.02, 3.15)	1.97 (1.39, 2.80)	4.21 (2.47, 7.19)	2.53 (1.77, 3.62)	0.066
RX opioids	1.20 (0.79, 1.83)	1.94 (0.84, 4.46)	1.10 (0.58, 2.08)	0.80 (0.26, 2.42)	0.398
Normative disapproval beliefs—overall	b	b	b	b	
Alcohol	0.62 (0.50, 0.77)	0.74 (0.51, 1.07)	0.56 (0.38, 0.82)	0.58 (0.41, 0.83)	0.518
Marijuana	0.29 (0.23, 0.38)	0.39 (0.26, 0.58)	0.16 (0.09, 0.30)	0.31 (0.21, 0.47)	0.069
RX opioids	0.84 (0.54, 1.32)	0.93 (0.36, 2.39)	0.62 (0.31, 1.24)	1.08 (0.47, 2.47)	0.580
Self-efficacy to refuse alcohol and drugs	0.54 (0.45, 0.64)	0.60 (0.45, 0.79)	0.34 (0.23, 0.51)	0.62 (0.48, 0.81)	**0.034**
Perception of getting in trouble for alcohol and drug use	0.59 (0.50, 0.70)	0.65 (0.50, 0.86)	0.54 (0.39, 0.75)	0.57 (0.43, 0.74)	0.643
Normative estimates of peer alcohol and drug use	1.50 (1.26, 1.80)	1.18 (0.88, 1.59)	1.63 (1.17, 2.27)	1.86 (1.35, 2.57)	0.102

a: Group specific ORs were not estimated due to lack of confirmation of scale invariance

b: overall and group specific ORs not estimated due to poor model fit in the overall sample

*p*-values in bold: < 0.05

*PHQ-8* Patient Health Questionnaire depression scale-8, *GAD-7* Generalized Anxiety Disorder Scale-7, *RX* prescription

## Data Availability

Underlying primary data will be made available to outside individuals via written request to the Emory University PIs (Livingston and Komro) and with approval of a data sharing agreement with the Cherokee Nation.
